# Exploring the impact of population ageing on the spread of emerging respiratory infections and the associated burden of mortality

**DOI:** 10.1186/s12879-023-08657-3

**Published:** 2023-11-07

**Authors:** Signe Møgelmose, Karel Neels, Philippe Beutels, Niel Hens

**Affiliations:** 1 Data Science Institute, Interuniversity Institute of Biostatistics and statistical Bioinformatics, Hasselt University, Hasselt, Belgium; 2 Center for Population, Family and Health, University of Antwerp, Antwerp, Belgium; 3 Centre for Health Economic Research and Modelling Infectious Diseases, Vaccine & Infectious Disease Institute, University of Antwerp, Antwerp, Belgium; 4 School of Public Health and Community Medicine, University of New South Wales, Sydney, Australia

**Keywords:** Demographic change, Population ageing, Disease burden, Infectious disease modelling, Epidemiology, Emerging infectious diseases

## Abstract

**Background:**

Increasing life expectancy and persistently low fertility levels have led to old population age structures in most high-income countries, and population ageing is expected to continue or even accelerate in the coming decades. While older adults on average have few interactions that potentially could lead to disease transmission, their morbidity and mortality due to infectious diseases, respiratory infections in particular, remain substantial. We aim to explore how population ageing affects the future transmission dynamics and mortality burden of emerging respiratory infections.

**Methods:**

Using longitudinal individual-level data from population registers, we model the Belgian population with evolving age and household structures, and explicitly consider long-term care facilities (LTCFs). Three scenarios are presented for the future proportion of older adults living in LTCFs. For each demographic scenario, we simulate outbreaks of SARS-CoV-2 and a novel influenza A virus in 2020, 2030, 2040 and 2050 and distinguish between household and community transmission. We estimate attack rates by age and household size/type, as well as disease-related deaths and the associated quality-adjusted life-years (QALYs) lost.

**Results:**

As the population is ageing, small households and LTCFs become more prevalent. Additionally, families with children become smaller (i.e. low fertility, single-parent families). The overall attack rate slightly decreases as the population is ageing, but to a larger degree for influenza than for SARS-CoV-2 due to differential age-specific attack rates. Nevertheless, the number of deaths and QALY losses per 1,000 people is increasing for both infections and at a speed influenced by the share living in LTCFs.

**Conclusion:**

Population ageing is associated with smaller outbreaks of COVID-19 and influenza, but at the same time it is causing a substantially larger burden of mortality, even if the proportion of LTCF residents were to decrease. These relationships are influenced by age patterns in epidemiological parameters. Not only the shift in the age distribution, but also the induced changes in the household structures are important to consider when assessing the potential impact of population ageing on the transmission and burden of emerging respiratory infections.

**Supplementary Information:**

The online version contains supplementary material available at 10.1186/s12879-023-08657-3.

## Background

The population age structures in most high-income countries have for decades been shifting towards older ages (i.e. population ageing) as a result of increasing life expectancy and persistent below-replacement fertility levels. Currently, a temporary acceleration of population ageing is seen in many countries due to the ageing of the large generations born in the mid-twentieth century [1]. Moreover, population ageing has become a global phenomenon and the proportion of older adults in many low- and middle-income countries is increasing at an unprecedented speed [2–5].

The rising burden of non-communicable diseases induced by population ageing has rightfully been given a lot of attention [6–8]. However, morbidity and mortality due to infectious diseases, respiratory infections in particular, remain substantial in the elderly population [9]. The progressive deterioration of immune functions with age (immunosenescene) increases older adults’ susceptibility to infection and their risk of a severe outcome in case of disease [10]. The COVID-19 pandemic, for example, has had a disproportionate impact on the older adult population and on those living in long-term care facilities (LTCFs) in particular [11–15]. Several aspects of LTCFs (e.g. communal meals, group activities, staff rotation) make them an optimal environment for rapid spread of many infectious diseases [16, 17]. Additionally, many LTCF residents have underlying chronic illnesses, which, in addition to their old age, may increase the severity of an infection [18, 19]. Nevertheless, LTCF residents only make up a minority of the older adult population in most high-income countries. The majority of old people typically live alone or with a partner. Moreover, social contact surveys from several European countries have shown that people aged 65 and older have the lowest mean number of contacts [20], and thus fewer interactions that potentially could lead to disease transmission. Consequently, the incidence of infections transmitted via close contact may be relatively low in the oldest age groups, yet the disease burden is typically substantial [21–25].

This implies that high-income countries with ageing populations may face a decreasing overall incidence of an infectious disease (e.g. influenza), but it could coincide with an increasing burden. However, the future burden of infectious diseases in older adults may, among other things, be influenced by the future health and living arrangements at old age. It remains unclear whether the increases in life expectancy are accompanied by a proportionate increase in healthy life expectancy [26–29]. Health at old age and living arrangements are naturally connected, with relevance for infections transmitted via close contact. The proportion of elderly people living with a partner is expected to increase due to improved longevity, particularly of males [30]. Nevertheless, the proportion living in LTCFs is also likely to increase as the proportion of the oldest people (i.e. 85+) increases [31, 32].

Several studies have investigated the impact of population ageing on the spread and burden of different infectious diseases, including measles, influenza, pneumonia and herpes zoster (e.g. [33–41]). Nevertheless, only few studies consider a household-structured population and to our knowledge none of them incorporate LTCFs. We aim to improve the understanding of how changing age and household structures affect the future transmission dynamics and mortality burden of respiratory infections in an ageing population, and explicitly explore the role of living arrangements in the older adult population. Specifically, we consider the Belgian population, which, like most other high-income countries, has a relatively old age structure and is still ageing. We use a demographic microsimulation, which is based on longitudinal microdata drawn from Belgian census and population registers [42]. The microsimulation includes dynamic demographic processes for fertility, mortality, migration and household transitions, making it possible to model the Belgian population over time with evolving age and household structures. In addition to private households, collective households (e.g. LTCFs) are represented in the microsimulation. Due to the uncertainty surrounding the future health and living arrangements of older adults, we consider three demographic scenarios with respect to the proportion of LTCF residents in the population. We refer to the scenarios as *low, medium* and *high* to describe the proportion of older adults living in LTCFs relatively to the other scenarios.

We subsequently combine the demographic microsimulation with a disease transmission model representing the spread of SARS-CoV-2 and a novel influenza A virus. The model is a two-level mixing model, which distinguishes between exposure to infection in the household and exposure in the community at large [42]. Additionally, the model implements contact networks within households which are based on empirical data [43]. We simulate outbreaks of SARS-CoV-2 and influenza in a fully susceptible population in 2020, 2030, 2040 and 2050, which allows sufficient time for demographic change to emerge.

We first illustrate how the age and household structures are altered in an ageing population. Secondly, we explore how the changing population structures affect the spread of the two respiratory infections (i.e. incidence) and the burden of mortality in the form of disease-related deaths and quality-adjusted life-years (QALYs) lost. In health economics, QALY expectations (gains or losses) represent a commonly used summary measure of longevity adjusted for the combined impact of death and morbidity [44]. Finally, we investigate to what extent our findings at the individual and population level are affected by changes in the living arrangements in the older adult population.

## Methods

We model the host population at the individual level using a demographic microsimulation for Belgium, which is combined with a disease transmission model to simulate the spread of an infection. Each individual is represented by a set of attributes, including age, sex, household membership and disease state. In each time step (i.e. day), the individuals’ attributes may change due to demographic events and events resulting from interactions between individuals (e.g. birth, leaving parental household, union formation, death, social contact and disease transmission). The population is thus evolving over time and demographic changes and disease outbreaks emerging at the population level can be tied to individual events and interactions between individuals.

### Demographic microsimulation

We simulate the Belgian population using the microsimulation presented in [42]. The initial population is made up by a household-based sample from the Belgian census in 2011 corresponding to about 10% of the population. For each individual, we have information on their date of birth, sex, ID of parents, birth trajectory (parity and date of most recent birth if applicable), household ID and household position (e.g. in union, child, single parent). Individuals can thus be linked to each other through household membership and kinship. The population evolves over time as individuals can enter and leave the population as a result of births, deaths and migration. Moreover, individuals can move between or create new households, for example as a part of union formation or dissolution. Finally, all individuals are ageing over time and the population is updated accordingly.

The probability of a demographic event taking place varies by individual characteristics, including age, sex and household position, and changes over time except for the household transition rates. We assume that the mortality, fertility and migration levels in the microsimulation are similar to the observed and projected rates by the Belgian Statistical Office (Statbel) and the Belgian Federal Planning Bureau (FPB). This implies below-replacement fertility (a total fertility rate below 2.1 [45]) and continuous improvements in longevity, especially for males [46, 47] (see Figs. S1 and S2 in Additional file 1). Consequently, the population will continue ageing, with implications for the household structures. Fertility trajectory and/or household position are included as covariates in the sub-models for fertility and mortality, as they have been shown to affect the probability of having a(nother) child and dying, respectively [48, 49]

We consider three demographic scenarios pertaining to the household structures in the older adult population (i.e. people aged 75 and older). The large majority of LTCF residents live in a single-person household prior to moving to the LTCF, thus we created three scenarios by varying the probability of leaving a single-person household for people aged 75 and older. The cut-off is made at the age of 75 years since only a small proportion of the population reside in LTCFs at younger ages (see Fig. S3 in Additional file 1). We refer to the scenarios as *low, medium* and *high*, as an indication of the proportion of the older adults living in LTCFs. The demographic data, model and source code are described in detail in [42] and section 1 in Additional file 1.

### Disease transmission model

In addition to the demographic attributes, all individuals are assigned a disease state. Disease outbreaks take place in the simulated population in 2020, 2030, 2040 and 2050 as ten randomly chosen individuals become infected, in an otherwise fully susceptible population, on January 1st of each respective year. The disease states are thus reset to *susceptible* and immunity obtained in a prior outbreak is disregarded as a new outbreak begins. The outbreaks are ten years apart to give sufficient time for demographic changes to emerge. We use an SEIR-like (*Susceptible-Exposed-Infectious-Recovered*) model to describe the spread of respiratory diseases transmitted via close-contact interactions with the examples of COVID-19 and influenza. The probability of becoming infected, and thus moving from the susceptible to exposed state, is calculated using a two-level mixing model, where an individual can become infected as a result of disease transmission within the household or in the general population [50].

#### Within- and between-household interactions

We use the same techniques as described in [42] to model social interactions, which serve as a proxy for an at-risk event at which infection can be transmitted. Contacts between non-household members in the general population are estimated using social contact data collected in a survey in Belgium in 2010-2011 [20] and made available as a contact matrix through the SOCRATES data tool [51] (see Fig. S6 in Additional file 1). Contacts between household members were excluded, as these are captured by the household level of the model, but contacts taking place in the household with non-household members were included. Additionally, supplementary professional contacts (SPC) were excluded. SPC is a category for individuals with more than 20 professional contacts per day (e.g. bus drivers). These are likely to be less important than other types of contacts when it comes to the transmission of close-contact infectious diseases [52].

For each household, we construct a contact network to model interactions among household members. Contacts are determined to take place using an exponential-family random graph model developed by Krivitsky et al. [43], which was fitted to data from the social contact survey mentioned above [20] and a household contact survey [53], both conducted in Belgium in 2010-2011. The household contact network is, amongst other things, conditional on the type of household and the age-sex composition. In each time step (i.e. day), we apply the fitted model from Krivitsky et al. [43] to generate a contact network for each household in the simulated population. The household contact networks may thus change every day. The mean network density (i.e. the number of links in a household relative to the number of possible links [54]) by household size and type are shown in Fig. S7 in Additional file 1.

#### Influenza

We formulate an SEIR model to describe the spread of a novel influenza virus such as the influenza A (H1N1)pdm09 virus that emerged in 2009. When acquiring the infection, the individual is not infectious at first (i.e. exposed or latent period), but becomes infectious as the latent period ends and eventually recovers as the infectious period comes to an end. Disease-related mortality is not considered explicitly in the model, but estimated after the simulation. Each susceptible individual *i* acquires the infection at time *t* with the probability:

1 $$\begin{aligned} p_i(t) = 1-\prod _{\begin{array}{c} j\ne i\\ j\in h_i \end{array}} \left( 1-\beta _ha_{ij}(t)I_j(t)\right) \cdot \prod _{j\notin h_i}\left( 1-\beta _pc_{ij}(t)I_j(t)\right) , \end{aligned}$$

where $h_i$ denotes the household of individual *i* and the parameters $\beta _h$ and $\beta _p$ represent the probability of disease transmission given contact between a susceptible and infectious individual within the household and in the general population, respectively. We select transmission parameters, $\beta _h$ and $\beta _p$, that result in a group-to-group reproduction number ($R_*$ [50]) of about 1.5, which resembles the basic reproduction number estimated for influenza A(H1N1)pdm09 [55–57]. This is further described in section 6 and 7 in Additional file 1. $I_j(t)$ takes the value one if individual *j* is infectious at time *t* and is otherwise zero. The contact network in household $h_i$ is represented by an adjacency matrix *A* and the element $a_{ij}(t)$ equals one if household members *i* and *j* come into contact with each other at time *t* and is otherwise zero. A new adjacency matrix is generated in each time step.

The social contact matrix from Fig. S6 in Additional file 1 contains the mean number of contacts per day in the general population between each age group, $m_{ij}$, and the probability that individual *i* and *j* come into contact with each other at time *t* given the age groups to which they belong, $c_{ij}(t)$, is calculated as follows:

2 $$\begin{aligned} c_{ij}(t)=\dfrac{m_{ij}}{N_j(t)}, \end{aligned}$$

The element $m_{ij}$ is divided by $N_j(t)$, the size of the age group of *j* at time *t*, to keep the age-specific contacts constant over time. This implies that we assume disease transmission in the general population to be frequency-dependent, meaning that the number of effective contacts made by each person remains unchanged as the population grows. In each time step, the probability of infection is computed for all susceptible individuals in the population and their disease state is updated accordingly.

The latent period is drawn from a uniform distribution with 1 day as minimum and 5 days as maximum. We assume that the infectious period follows a gamma distribution with a mean of 3.8 days and standard deviation of 2 days [58–60]. For each newly infected individual, a value is drawn from the distribution and rounded to the nearest integer. An infected individual recovers and obtains immunity when the infectious period has passed.

#### COVID-19

**Fig. 1:**
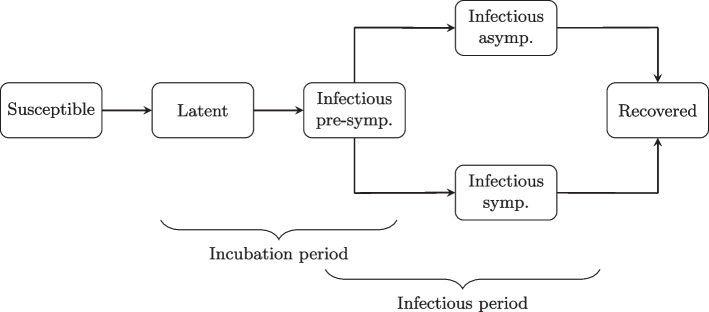
Disease transmission process for COVID-19. Symp.: Symptomatic

In order to model the spread of SARS-CoV-2, we use a model similar to [61], which involves an extension of the SEIR model. Infectious individuals are initially pre-symptomatic and some develop symptoms while others remain asymptomatic (see Fig. 1).

Each susceptible individual *i* acquires infection at time *t* with probability:

3 $$\begin{aligned} p_i(t) = 1&- \prod _{\begin{array}{c} j\ne i\\ j\in h_i \end{array}} \left( 1-\beta _{h,a}a_{ij}(t)I_{j,a}(t)s_i\right) \cdot \prod _{\begin{array}{c} j\ne i\\ j\in h_i \end{array}} \left( 1-\beta _{h,s}a_{ij}(t)I_{j,s}(t)s_i\right) \nonumber \\&\quad \cdot \prod _{j\notin h_i}\left( 1-\beta _{p,a}c_{ij}(t)I_{j,a}(t)s_i\right) \cdot \prod _{j\notin h_i}\left( 1-\beta _{p,s}c_{ij}(t)I_{j,s}(t)s_i\right) . \end{aligned}$$

The same notation is used as for the influenza model, but the subscripts indicate whether the infectious individual is symptomatic (s) or asymptomatic (a). Infected individuals without symptoms are assumed to be half as infectious compared to those with symptoms, however, we acknowledge that this parameter is associated with uncertainty [62, 63]. We select transmission parameters, $\beta _h$ and $\beta _p$, that result in a group-to-group reproduction number of about 3, to reflect the estimated basic reproduction number in Belgium prior to lockdown [61, 64, 65]. This is further described in section 6 and 7 in Additional file 1.

$I_{j,a}(t)$ ($I_{j,s}(t)$) takes the value one if individual *j* is infectious and asymptomatic (symptomatic) at time *t* and is otherwise zero. The parameter $s_i$ represents age-specific susceptibility and is 0.5 if individual *i* is younger than 18 years of age and is otherwise one, as we assume that children and teenagers are half as susceptible as adults [66]. The incubation period contains a latent period and a pre-symptomatic period. In the latent period, the individual is infected but not yet infectious, whereas the individual is infectious in the pre-symptomatic period, but shows no symptoms (yet). The incubation period is based on findings from [62, 67] and is assumed to follow a log-normal distribution with mean and standard deviation on the log scale of 1.43 and 0.66, respectively (see Fig. S10 in Additional file 1). The incubation period spans over at least two days since we assume that infectiousness starts one day prior to symptom onset at the latest and one day after infection at the earliest.

Based on infectiousness profiles from [67], a discrete distribution for the pre-symptomatic infectious period was estimated in [61] (see Fig. S11 in Additional file 1). For each newly infected individual, we draw from the distributions for the incubation period and the pre-symptomatic period. The length of the latent period for a given individual is obtained by subtracting the sampled value for the pre-symptomatic period from that of the incubation period (after rounding to a discrete number of days).

The distribution of the infectious period (including pre-symptomatic period) is assumed to follow a normal distribution with a mean of six days and a standard deviation of one (see Fig. S12 in Additional file 1). For each infected individual, the length of the infectious period is drawn from the distribution and the pre-symptomatic period is subtracted in order to obtain the remaining days of infectiousness. It is determined whether the individual shows symptoms during this period according to age-specific probabilities estimated in [61] (see Fig. S13 in Additional file 1). The probability of being symptomatic is based on the age-specific relative susceptibility to symptomatic infection reported in [68] assuming 50% of the overall cases in the population to be symptomatic. An infected individual recovers and obtains immunity when the infectious period has passed. We run 50 simulations using the COVID-19 and influenza models, respectively, but limit our analysis to those simulations where an outbreak takes place (i.e. total attack rate of at least 0.5%).

#### Disease-related mortality

We estimate influenza-related deaths by applying the infection fatality rates (IFRs) for the influenza A(H1N1)pdm09 pandemic estimated by Riley et al. [69] based on a serological survey of a cohort of households in Hong Kong (see Fig. S14 in Additional file 1). The rates are by age group, but no estimates are available for children younger than three years. Consequently, we apply the IFR of the age group 3-19 to all ages younger than 19, although this is likely to underestimate the fatalities in the youngest children. For COVID-19, we use IFRs estimated by Molenberghs et al. [15] for Belgium in the period March 8th to May 9th 2020. The IFRs are broken down by age and household type (see Fig. S15 in Additional file 1). The considered household types are LTCFs and non-LTCFs. For ages younger than 60 years, there is no distinction between the household types, likely due to the small number of LTCF residents of that age. LTCF residents are not directly identifiable in the demographic microsimulation. Therefore, we use the household position *collective* as a proxy. This household position covers residents in different types of institutions, including prisons and LTCFs, but we expect the large majority of older adults with that household position to actually be living in LTCFs.

#### Quality-adjusted life years

To provide an estimate of the potential years of life lost due to premature death and the health-related quality of those years of life lost, we estimate the QALY losses attributable to COVID-19 and influenza fatalities using the method presented by Briggs et al. [70]. Pre-existing comorbidities are associated with an increased risk of a fatal outcome upon infection with SARS-CoV-2 or influenza [71–73], which is taken into account when estimating the QALY losses (see further details in section 10 in Additional file 1). However, we do not consider QALY losses from morbidity due to non-fatal infections.

## Results

### Population ageing

**Fig. 2:**
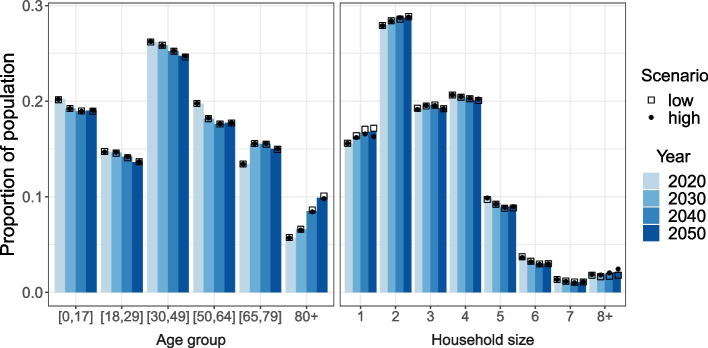
Age and household-size distribution by simulation year and scenario (medium: bar, low: square, high: circle)

The population is ageing during the whole simulation period. Between 2020 and 2030, it is primarily due to an increasing proportion aged 65-79 years, while the age group 80 years and older increases fastest in the remaining decades (see Fig. 2 left panel). This reflects the ageing of the large generations born in the mid-twentieth century. Population ageing induces changes in the household size distribution (see Fig. 2 right panel). The elderly population primarily lives in small households (size 1-2) or very large households in the form of LTCFs (see Fig. S3 in Additional file 1). Consequently, an increasing proportion of the population lives in households of these sizes (1, 2 and 8+) as the population is ageing. Additionally, households of nuclear families are decreasing in size due to low fertility and an increase in single-parent families. It should be noted that the group size 8+ primarily is made up by LTCFs, which tend to have 25-100 residents in the simulated population.

The differences in the demographic scenarios only slightly affect the age distribution. The proportion aged 80 years and older is marginally larger in the scenario *low* (square) than in *medium* (bar) and *high* (dot), because of the smaller proportion of LTCF residents, which have a higher all-cause mortality (for further information on all-cause mortality in the microsimulation see section 1 in Additional file 1). The demographic scenarios have a more profound impact on the household size distribution. The proportion in household size 1 and 8+ in scenario *low* (square) and *high* (dot) gradually diverge from the medium scenario (bar), but in opposite directions. Consequently, the proportion living in LTCFs (i.e. size 8+) relative to the proportion living alone is highest in the scenario *high* and lowest in scenario *low*, while the *medium* scenario is in between. The other household sizes are more or less unaffected. Household size distributions by age groups, scenario and simulation year can be seen in Figs. S4 and S5 in Additional file 1.

### Transmission dynamics

As expected, the proportion of the population that becomes infected during an outbreak (attack rate) in the COVID-19 model is substantially larger than for influenza (see Fig. S16 in Additional file 1), due to the differences in the transmission parameters. The attack rate is decreasing over time in both models, but after 2040, a slight increase is seen for influenza (see Fig. S17 in Additional file 1). Older adults, which are increasingly replacing the younger population, have relatively fewer contacts on average since the majority live in small households (see Fig. S3 in Additional file 1) and have fewer contacts in the general population (see Fig. S6 in Additional file 1). Consequently, the older adult population has a lower risk of acquiring and transmitting an infection than younger age groups.

**Fig. 3:**
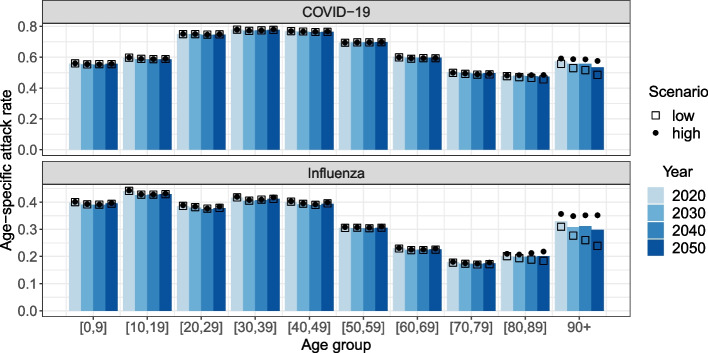
Mean age-specific attack rates by simulation year, model and demographic scenario. Note the different scales on y-axes

The age-specific attack rates in the COVID-19 and influenza models naturally differ in magnitude, but other patterns are also seen (see Fig. 3, note different scales on y-axes). For COVID-19 (Fig. 3, upper panel), the attack rate is largest in the adult population, which reflects the lower susceptibility of children and the increasing probability of being symptomatic and thereby more contagious with age. Meanwhile, influenza (Fig. 3, lower panel) is more prevalent in children and adolescents compared to adults, which is resulting from the age-specific differences in contact patterns within and outside the household. This indirectly affects the parental generation (i.e. ages 30-49), which has the highest incidence in the adult population. In both models, the attack rate in the elderly population is shaped by the proportion of the age groups living in LTCFs, as the risk of infection increases with household size (see Fig. S19 in Additional file 1).

These age patterns in the transmission processes imply that the impact of population ageing on the spread of COVID-19 and influenza differ. Since children and adolescents are the main drivers in influenza transmission, the attack rate declines substantially as the nuclear families decrease in size (i.e. low fertility and increase in single-parent families) and are increasingly being replaced by elderly people with a relatively low risk of infection (see Fig. S17 in Additional file 1). Meanwhile, the decrease in the attack rate of COVID-19 is less pronounced, and barely observed for the 20- to 79-year-olds (see Fig. S18 in Additional file 1). The risk of community transmission in the young and middle-aged adult population remains substantial (i.e. at work-places) due to the increased probability of being symptomatic and the assumption of frequency-dependent transmission. Moreover, old people (i.e. 70+) account for a larger share of infections with COVID-19 than with influenza, thus the decrease in the overall attack rate of COVID-19 induced by population ageing is less pronounced.

**Fig. 4:**
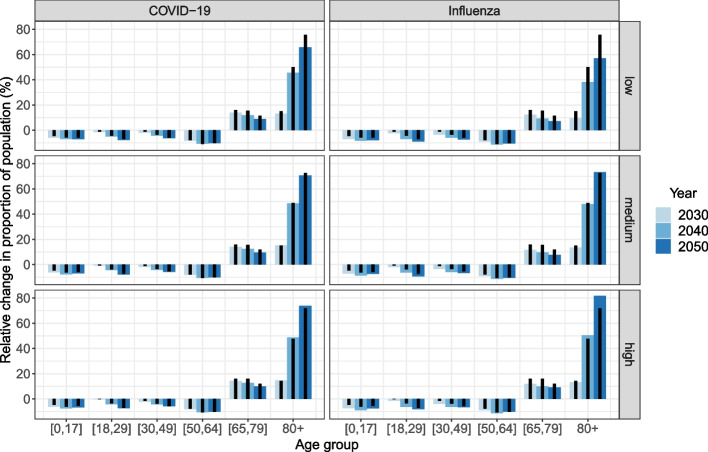
Mean relative change in size of age group (black bars) and number of infected people in age group (blue bars) as proportion of total population compared to 2020. Demographic scenarios by row and models by column

Since the population is ageing, the age composition of the infected people in the population is also shifting, but not necessarily to the same degree. In Fig. 4, we compare the relative change in the age distribution (black bars) to the relative change in the age distribution of infected people (blue bars), both as a proportion of the total population size in simulation year 2030, 2040 and 2050 and using the corresponding values for 2020 as the reference. Infected people younger than 65 years of age make up a decreasing proportion of the population, while the proportion of infected people aged 65 and older is increasing, as to be expected considering the changes in the age distribution. The proportion of infected children and adolescents (i.e. younger than 18 years) in the population is decreasing more than the overall proportion of the age group across all demographic scenarios and models. This results from the decreasing household size of nuclear families (see Fig. S4 in Additional file 1), which is associated with a lower risk of infection for children and their parents as described earlier. Nevertheless, in the COVID-19 model, young and middle-aged adults experience a more or less equal relative change in the proportions since community transmission is more pronounced. This is also the case for age group 50-64 in the influenza model, but due to an increasing mean household size as the share living together with their adult children is increasing. The growth in the proportion of infected 65 to 79-year-olds in the population is slower than the overall growth in the age group, as the share living in single-person households is increasing, especially for people in their seventies.

The relationship between the growth in the proportion of infected people aged 80 and older in the population and the general growth in the age group vary by demographic scenario. In the medium scenario, the growth rates are very similar, meaning that the age group is barely benefitting from the lower transmission in the young population. First of all, older adults have few contacts with children and adolescents. Second of all, the share of the age group 80+ living with a partner instead of alone is increasing. Finally, the age group is increasingly made up by people aged 90 and older, which have a higher attack rate.

In the scenario with a relatively low and decreasing share of the population living in LTCFs (first row in Fig. 4), the proportion of infected people aged 80 years and older in the population is growing at a slower rate than the age group overall, while this relationship is reversed in the scenario with a high and slightly increasing share living in LTCFs (third row in Fig. 4). The differences between the scenarios *low* and *high* for age group 80+ are generally larger in the influenza model than in the COVID-19 model. The risk of infection for an elderly person living in a small household compared to someone living in an LTCF differs substantially more in the influenza model than in the COVID-19 model, thus the response to the scenarios is more pronounced in the first case (see Fig. S20 in Additional file 1). This is again related to the age-specific susceptibility and infectiousness in the COVID-19 model.

### Burden of disease-related mortality

**Fig. 5:**
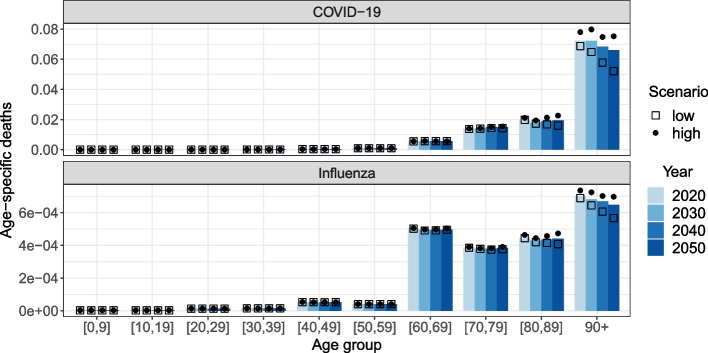
Mean age-specific disease-related death rate by year, model and demographic scenario. Note the different scales on y-axes

Although the overall attack rate is decreasing over time, the number of deaths per 1,000 people in the population is increasing, due to the shift in the age distribution of the infected population (see Fig. S21 in Additional file 1). Since the applied fatality rates are associated with substantial uncertainty, we limit the analysis of disease-related deaths to a comparison across time, age and demographic scenarios. Deaths attributable to COVID-19 are highly concentrated in the older adult population (see Fig. 5, upper panel). Influenza-related deaths are also more pronounced in the older adult population, however, differences within the older age groups only reflect differential attack rates since the same IFR is applied to everyone aged 60 and older. The differences in fatalities between the demographic scenarios are induced by the aforementioned relationship between the proportion of infected older adults and the proportion of those living in LTCFs. Moreover, the applied IFRs for COVID-19 from [15] are broken down by household type (i.e. LTCF vs. non-LTCF), hence the number of COVID-19 deaths in our simulation is more sensitive to changes in the population living in LTCFs.

**Fig. 6:**
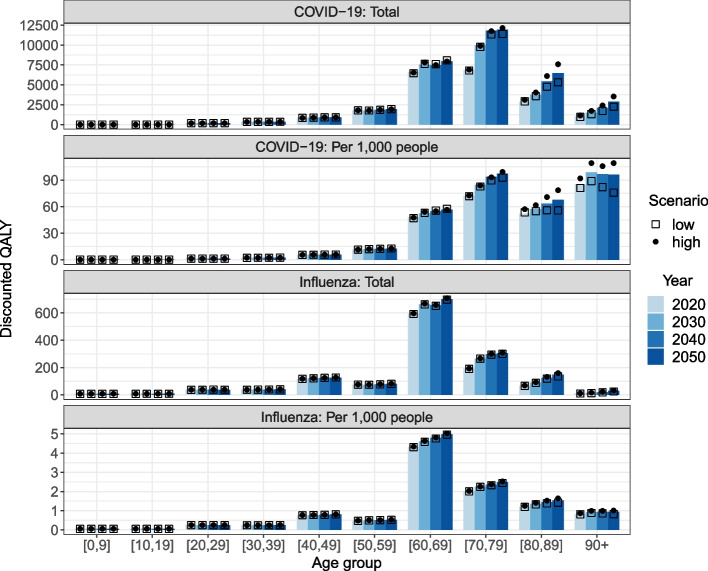
Mean age-specific QALY losses in total and per 1,000 people by year and demographic scenario

Clearly, the average number and quality of years of life lost due to a premature death decrease with age. The middle-aged adults thus account for a larger share of the total QALY losses than of the fatalities (Fig. 6, row one and three, note different Y-axis scales). However, the largest QALY losses in absolute values are seen in the 60-79 year olds for both COVID-19 and influenza. When taking the age distribution into account, the QALY losses become more pronounced in the oldest age groups (see Fig. 6, row two and four). In both models, the total QALY losses are increasing over time and at a rate slightly higher than that of the increase in deaths (see Fig. S21 in Additional file 1). Moreover, the age-specific QALY losses per 1,000 people are increasing in several age groups despite a stable or even decreasing disease-related death rate (see Fig. 6), because life expectancy is increasing. In 2020, for example, the average life expectancy of a 75 year old is about 12 years, while it is expected to increase to 15 years by 2050 (see Fig. S22 in Additional file 1). However, the COVID-19 related QALY losses per 1,000 people in age group 90+ is decreasing between 2030 and 2050 for the demographic scenarios with a low or medium proportion of older adults living in LTCFs. The increasing life expectancy in this age group cannot compensate for the decrease in deaths associated with the changing living arrangements.

## Discussion

Increasing life expectancy and persistently low fertility levels have led to old population age structures in most high-income countries, and population ageing is ongoing as the large generations born in the mid-twentieth century move into the older age categories [1]. Population ageing has potential implications for the burden of infectious diseases as the morbidity and mortality of many infections are concentrated in the older adult population [9, 10], as seen in the COVID-19 pandemic. The demographic microsimulation and two-level mixing model applied in our study allow to investigate the potential impact of population ageing on the transmission dynamics and burden of COVID-19 and influenza, while explicitly considering changes in the household structures, particularly among the older adults. Our focus on the future living arrangements in the older adult population is motivated by the disproportionate burden of the COVID-19 pandemic among LTCF residents [11, 12, 14, 15].

Our results suggest that population ageing on the one hand is associated with smaller total attack rates in COVID-19 and influenza epidemics, but on the other hand is causing a substantially larger disease burden of mortality, even if the proportion of older adults living in LTCFs were to decrease. Moreover, we find that not only the shift in the age distribution, but also the induced changes in the household structures are important to consider when assessing the potential impact of population ageing on the transmission and burden of respiratory infections.

Respiratory infections are predominantly caught by close contact with an infectious individual and transmission often takes place between household members [74, 75]. Since older adults in Belgium have few contacts and the majority live alone or with their partner, the number of occasions where they could acquire a respiratory disease is relatively low. A decrease in the overall attack rate of COVID-19 and influenza is thus a logical consequence of population ageing. This relationship, however, is modified by the age pattern in the attack rates, which in turn is influenced by the susceptibility to and infectiousness upon infection as well as contact patterns.

In our simulation, COVID-19 attack rates were highest in the young and middle-aged adult population, while influenza incidence was highest in children and adolescents, which is in line with serological studies [76, 77]. Moreover, older adults made up a larger share of the infected population in the COVID-19 model than in the influenza model. Consequently, the increasing proportion of older people in the population led to a greater relative decline in the overall number of influenza infections, which was amplified by the decreasing size of nuclear families, as they play an important role in influenza transmission. The changing composition of nuclear families resulted from a decreasing fertility rate prior to 2020, which recovered slowly, but not fully, in the remaining simulation period, similar to observed and projected rates by Statbel and FPB [47]. Additionally, single-parent families became more prevalent. As a result of this, the average influenza incidence in children and the parental generation declined. This was less pronounced in the COVID-19 model, where the attack rate in the young and middle-aged adults were more or less unchanged due to substantial community transmission (i.e. at work-places).

Since older adults have the lowest number of community contacts [78], their risk of infection is highly dependent on their living arrangements. Older people in Belgium tend to either live in very small households (alone or with a partner) or in very large LTCFs. Several typical aspects of LTCFs, such as the size, shared meals, group activities, staff rotation, visitors, makes it easy for an infection to enter and spread rapidly [16, 17]. The variability in older adults’ risks of infection is thus considerably larger than in any other age group. This implies that the future incidence in the older adult population is closely connected to changes in their household structures.

In the microsimulation, single-person households (e.g. a widow) in the age group 80+ are increasingly being replaced by two-person households (e.g. a couple), as the sex differential in mortality diminishes due to larger improvements in the life expectancy of males than in that of females. On the one hand, living with a partner instead of living alone increases the risk of infection as household transmission becomes a possibility. On the other hand, the probability of moving to a LTCF, which is associated with a substantially higher risk of infection, is markedly lower for elderly people living with a partner than for those living alone. However, the future mortality, health and living arrangements in the older adult population are associated with a large degree of uncertainty [26–30, 79]. Therefore, we presented three demographic scenarios pertaining to the proportion of older adults living in LTCFs relative to the proportion living alone.

The attack rates in the old age groups follow the proportion living in LTCFs and therefore differ substantially between the demographic scenarios. However, the sensitivity of the attack rates to household structures among older adults was larger for influenza than for COVID-19. This is again related to the different age patterns in disease transmission. The risk of acquiring COVID-19 remains relatively high for older non-LTCF residents because their susceptibility is high and the few contacts they do have will typically be with other old people, which are most likely to be symptomatic in case of infection and thereby more contagious. The influenza attack rate in older non-LTCF residents is markedly lower than other population groups, as children and adolescents are the main drivers of the spread and rarely live together with old people and generally have few contacts with them. Meanwhile, the attack rate for LTCF residents of COVID-19 as well as influenza are the highest in the population. Thus, the larger differential in the influenza attack rates between LTCF and non-LTCF residents imply that the overall attack rate of the older adults responds stronger, in relative terms, to changes in the living arrangements.

Although population ageing is associated with a decreasing proportion of infected people in the total population, disease-related deaths and QALY losses are increasing substantially. The lost QALYs increase faster than the deaths because a projected increase in life expectancy is accounted for in the QALY estimations. The speed at which the burden increases is influenced by the living arrangements among older adults, which can be considered a proxy for the health at old age. However, even in a scenario with a diminishing proportion of LTCF residents, the burden of disease-related mortality increases considerably in the whole simulation period.

We emphasise that our study is an investigation of the effects of population ageing on transmission dynamics and burden of disease-related mortality, and the results should not be interpreted as predictions. Moreover, our findings should be seen in the light of several limitations. First, we restricted our study to emerging infectious diseases by assuming that the initial population is fully susceptible and we did not consider behavioural changes (e.g. changing contact patterns) during the outbreak, which would reduce the size of the simulated outbreaks. Additionally, age patterns of prior immunity or mitigation strategies in certain population groups (e.g. LTCFs, schools) may shift the age distribution of the infected population and thereby modify the impact of population ageing. For example, some degree of pre-existing immunity to influenza A (H1N1)pdm09 was found in older adults, which may have resulted from exposure to H1N1 viruses earlier in life [76]. Nevertheless, we disregard these elements in order to obtain a clear understanding of the effects of population ageing alone.

Second, we do not distinguish between locations at which social contacts take place outside the households (i.e. schools, workplaces) and the patterns of social contacts in the general population are assumed ot remain constant over time, since little is known about how contact patterns are affected by changing population structures. Nevertheless, the household contact patterns change along with the household composition as we generate new household networks in each time-step (i.e. day). The extrapolation of household contact networks for private households to LTCFs may be questionable due to the different structures, compositions and relations within the households. However, the large outbreaks among LTCF residents in our simulation reflect estimations of COVID-19 cases and the spread in LTCFs in Belgium prior to the implementation of mitigation measures [15, 80].

Third, the model for influenza was less detailed than that of COVID-19 due to differences in the availability of detailed epidemiological and clinical data, such as age-specific susceptibility and probability of developing symptoms.

Finally, our estimates of disease burden are based on adjusting the QALYs lost due to deaths attributable to COVID-19 and influenza, but do not include QALY losses from morbidity due to non-fatal COVID-19 or influenza. Furthermore, our QALY estimates are associated with a considerable degree of uncertainty pertaining to the applied IFRs and the parameter settings in the QALY estimations. Moreover, we applied constant IFRs and parameters in the QALY estimations. The alternative parameter settings suggested in Briggs et al. [70] for the estimation of QALYs did not change the relationships we obtained. Nevertheless, the age-specific morbidity and mortality associated with respiratory diseases may change over time because of medical innovations and/or improved health at old age. Developments in healthy life expectancy, however, remain unclear [26–29]. We partially addressed this uncertainty with the demographic scenarios in our analysis.

## Conclusions

Population ageing is associated with smaller outbreaks of emerging respiratory infections such as SARS-CoV-2 and novel influenza A virus. Nevertheless, the burden of mortality increases substantially, even if the population living in LTCFs, which typically face a high risk of infection as well as a fatal outcome, were to decrease. The variability in older adults’ risks of infection is considerably larger than in any other age group, which is related to their living arrangements. Not only the shift in the age distribution, but also the induced changes in the household structures are important to consider when assessing the potential impact of population ageing on the transmission and burden of emerging respiratory infections. Age patterns in epidemiological parameters may exacerbate or alleviate these relationships.

### Supplementary Information

**Additional file 1.** 

## Data Availability

The input data of this study are available from Statics Belgium but restrictions apply to the availability of these data, which were used under license for the current study, and so are not publicly available. A detailed description of the data and source code are available from the GitHub repository: https://github.com/signemoegelmose/demographic_microsimulation_EXTERNAL.

## Code availability

The source code is available from the GitHub repository: https://github.com/signemoegelmose/demographic_microsimulation_EXTERNAL.
