# Supplementary material for: Exploring the impact of population ageing on the spread of emerging respiratory infections and the associated burden of mortality
Source: BMC Infect Dis. 2023 Nov 7;23:767. doi: 10.1186/s12879-023-08657-3 (PMC10629067; doi:10.1186/s12879-023-08657-3)
Supplement: Supplementary file 1 — Additional file 1. [file 12879_2023_8657_MOESM1_ESM.pdf]

Additional file 1: Exploring the impact of population ageing on the spread of emerging respiratory infections and the associated burden of mortality

Contents

1 Demographic microsimulation 2

2 Trends in fertility and life expectancy (Statbel) 2

3 Population structures 3

4 Social contact matrix 6

5 Household network density 7

6 Threshold parameter R_* 8

7 Transmission parameters: β_h and β_p 8

8 Transmission parameters: COVID-19 10

9 Disease-related mortality 12

10 Estimation of QALY losses 13

11 Additional results 14

1 Demographic microsimulation

The GitHub repository below contains the source code for the demographic microsimulation, as well as a detailed description of the demographic data, model assumptions and implementation in the file *Documentation_data_and_microsimulation.docx*.

https://github.com/signemoegelmose/demographic_microsimulation_EXTERNAL

2 Trends in fertility and life expectancy (Statbel)

Figure S1 and Figure S2 display the total fertility rate and life expectancy at birth in Belgium, respectively, according to estimations (black) and projections (grey) by Statbel.

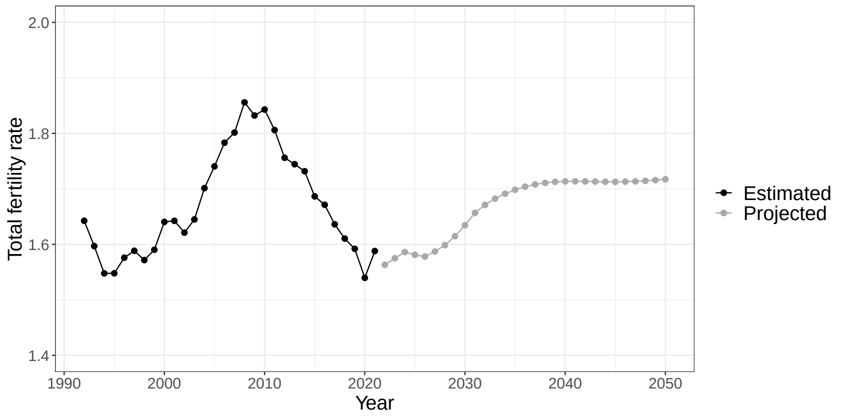

Fig. S1 Estimated and projected total fertility rate for Belgium, 1992-2050. Source: Statbel.

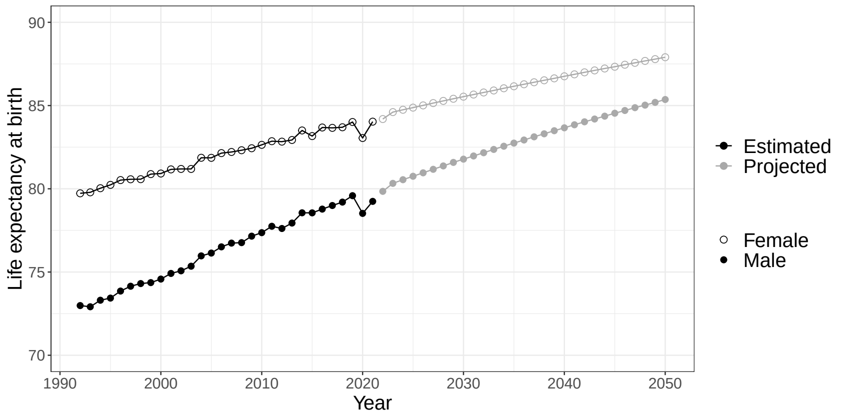

Fig. S2 Estimated and projected life expectancy at birth for Belgium, 1992-2050. Source: Statbel.

3 Population structures

The household size distribution is shown by age group in Figure S3 for the simulated population in 2020 in the scenario *medium* (i.e. scenarios referring to the share living in LTCFs).

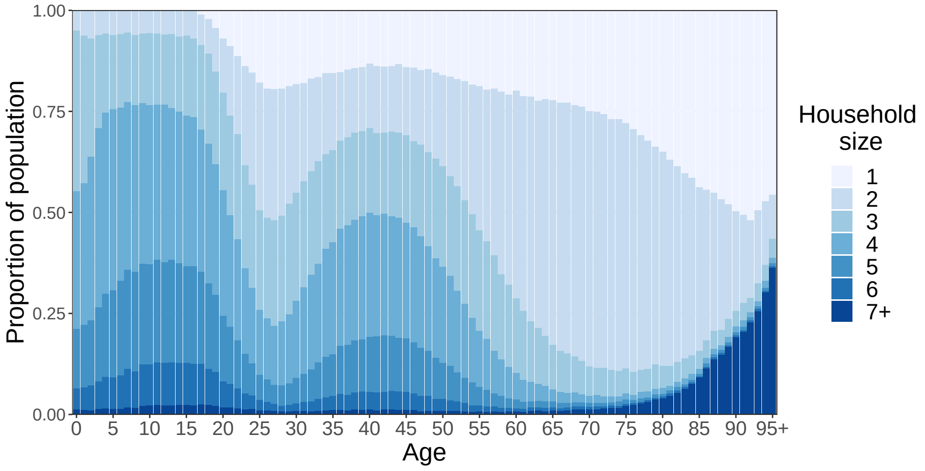

Fig. S3 Household size distribution by age group of simulated population in 2020. Medium scenario.

The household size distribution is shown by age group in Figure S4 for the simulated population over time (x-axis) in the scenario *medium* (i.e. scenarios referring to the share living in LTCFs).

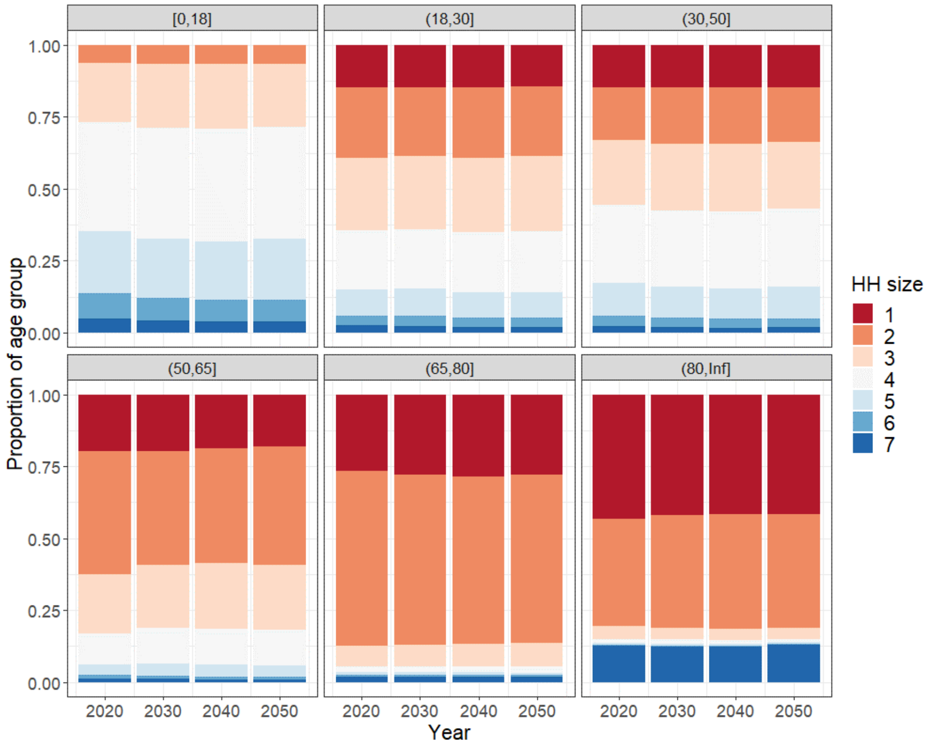

Fig. S4 Household size distribution by age group and year in medium scenario. 7=7+.

The household size distribution is shown by age group (rows) in Figure S5 for the simulated population over time (columns) and across scenarios (x-axis) (i.e. scenarios referring to the share living in LTCFs).

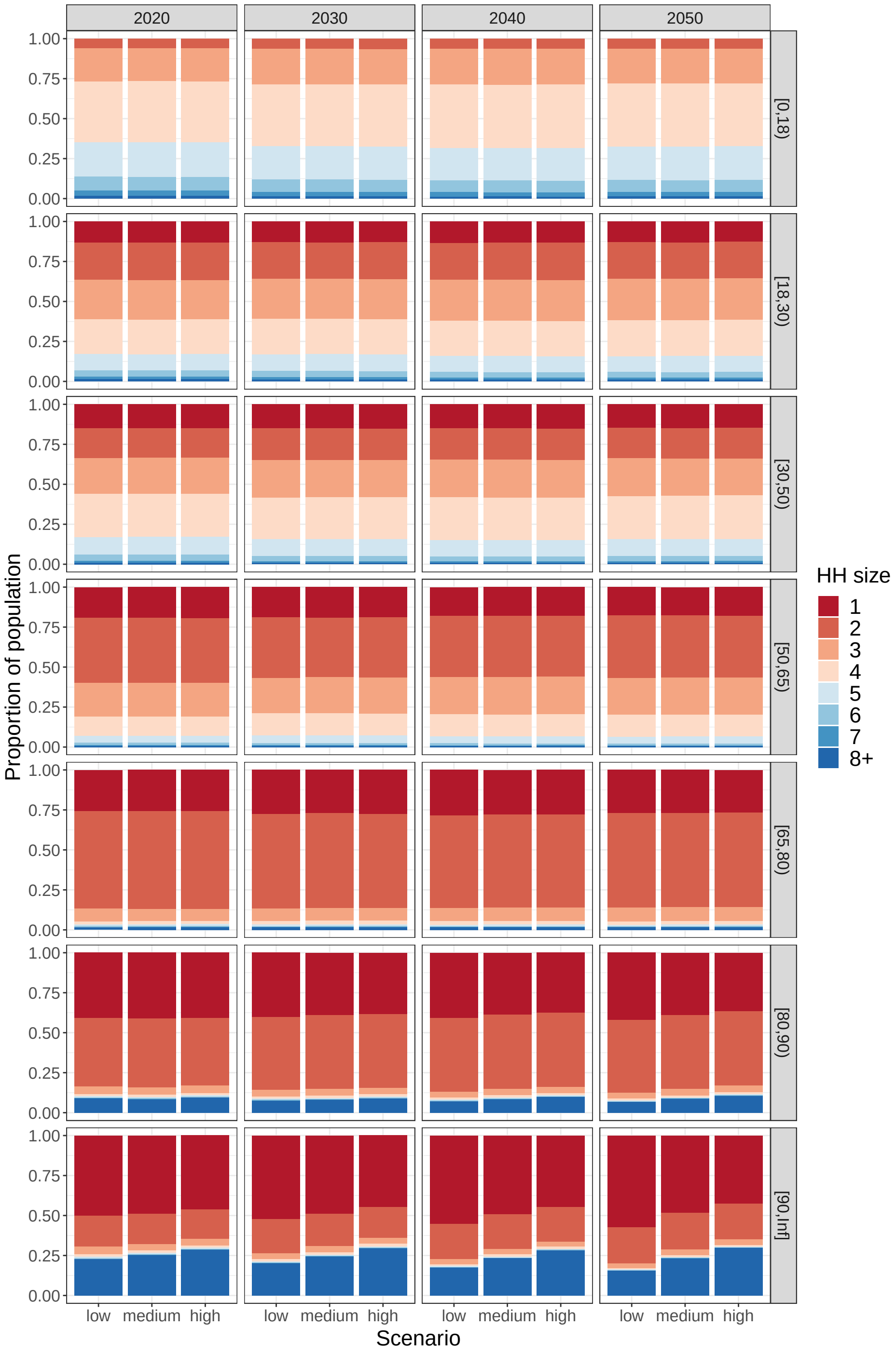

Fig. S5 Household size distribution by age, year and scenario.

4 Social contact matrix

The social contact matrix applied in the disease transmission model is visualised in Figure S6, where the colour indicates the average daily number of contacts between age groups.

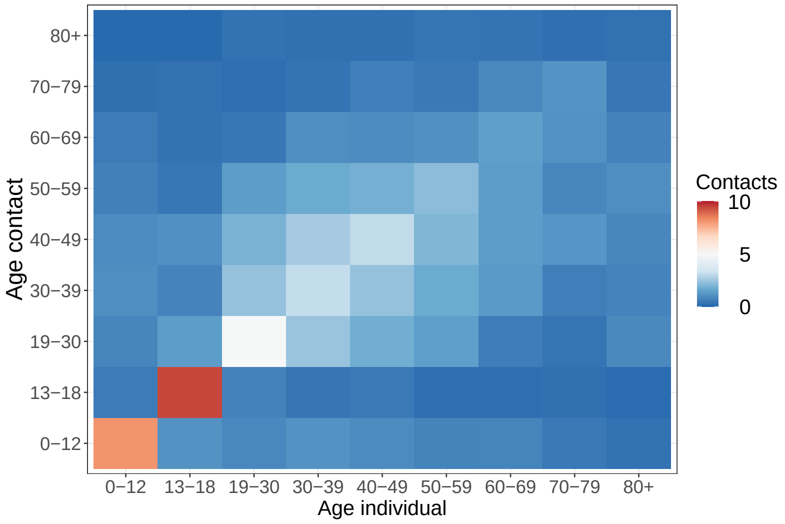

Fig. S6 Age-specific social contacts in Belgium excluding contacts with household members and excluding supplementary professional contacts [1, 2].

5 Household network density

The distribution of the household network densities (i.e. the number of links in a household relative to the number of possible links) by household size and type are shown in Figure S7, where the blue line indicates the overall mean, while the red and green dashed lines indicate the mean density for households with and without at least one child younger than 13 years, respectively.

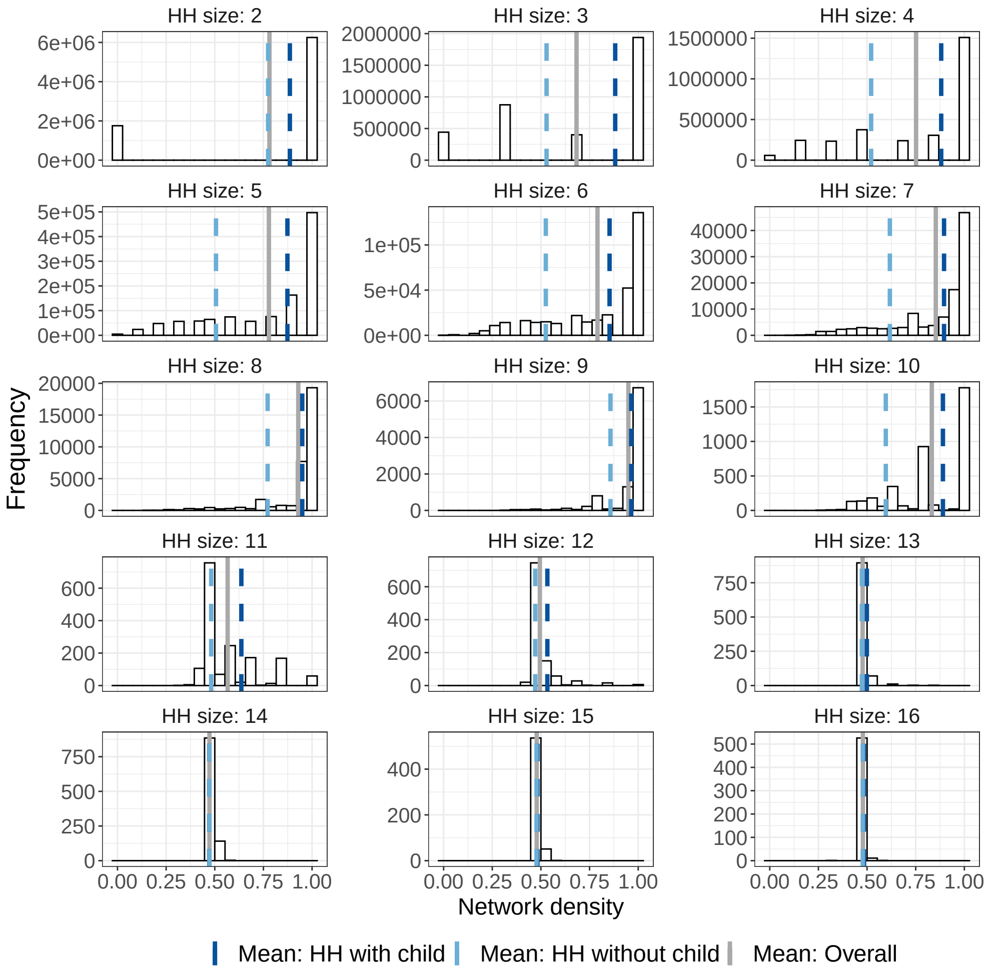

Fig. S7 Histograms of household network densities by household size (HH: household, child: age < 13).

6 Threshold parameter R_*

We compute the threshold parameter R_* (group-to-group reproduction number) based on [3]. The basic reproduction number R_0 is not used because it requires large group sizes, which is not the case for the households in our two-level mixing model. The computation of R_* is based on equation (3.31) in [3]:

$$R_* = \lambda_G E[T_I]\mu_h^{-1} \sum_{n=1}^{\infty} \left(1 + \mu_{n-1,1}\right) nh_n \tag{1}$$

$$R_* = \mu R_G \tag{2}$$

where n corresponds to household size, h_n is the proportion of households of size n and μ_h is the mean household size. We compute the average final size in households of size n, $(1 + \mu_{n-1,1})$, by starting with one randomly chosen infected individual in each household, which then can pass on the infection to household members, which also can transmit the infection within the household. Meanwhile transmission in the general population is disregarded. Finally, the average final size by household size is calculated and used to compute $\mu = \mu_h^{-1} \sum_{n=1}^{\infty} \left(1 + \mu_{n-1,1}\right) nh_n$ (i.e. the average number of household infections). The basic reproduction number in the general population when disregarding household transmission, $R_G = \lambda_G E[T_I]$, is computed by initially infecting one randomly chosen individual in the population. The individual can transmit the infection to others in the population, but the newly infected individuals cannot pass on the infection. The average number of secondary cases is then calculated.

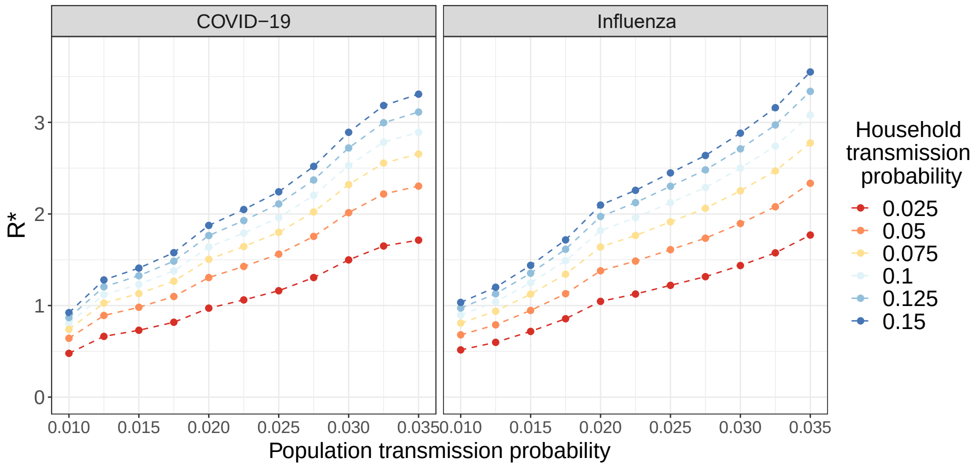

Fig. S8 R_* for medium demographic scenario in 2020.

7 Transmission parameters: β_h and β_p

We select the transmission parameters, β_h and β_p, based on the R_* in Figure S8 and the household secondary attack rates in Figure S9. In the model for COVID-19, we assume an R_*

of approximately 3 to reflect SARS-CoV-2 transmission in Belgium prior to the implementation of mitigation measures [4–6]. In the ILI model, we assume an R_* of approximately 1.5, which resembles the basic reproduction number estimated for the 2009 influenza A/H1N1 pandemic [7–9]. We choose the parameters $\beta_{h,s} = 0.125$ and $\beta_{p,s} = 0.0325$ for COVID-19 and $\beta_h = 0.075$ and $\beta_p = 0.02$ for ILI. With these parameters we obtain household secondary attack rates of approximately 0.34 and 0.19 for COVID-19 and ILI. This reflects estimated household secondary attack rates from several studies [10–12], however, these are associated with substantial variability.

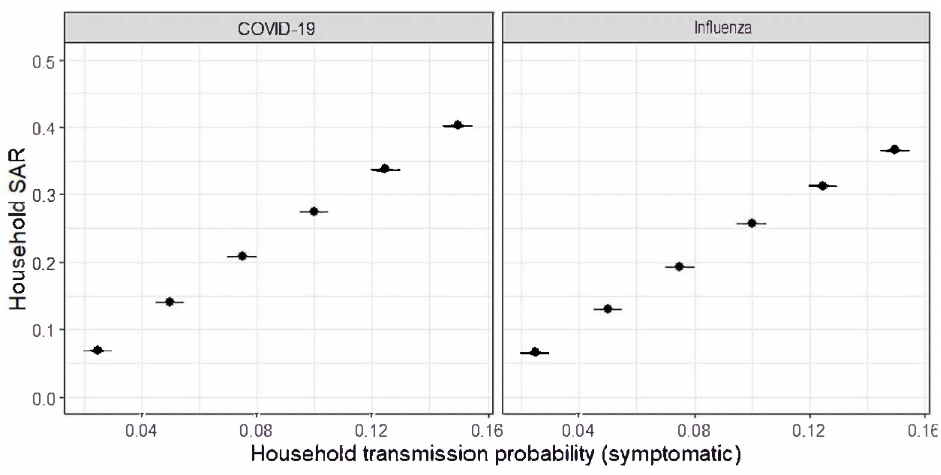

Fig. S9 Household secondary attack rate (household transmission only) for medium demographic scenario in 2020.

8 Transmission parameters: COVID-19

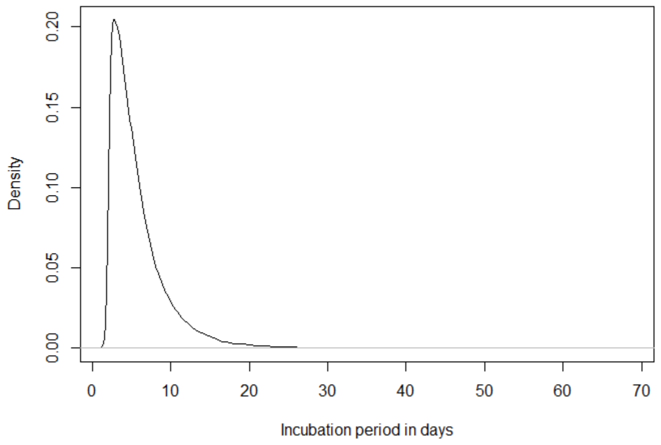

Fig. S10 Incubation period from [13]

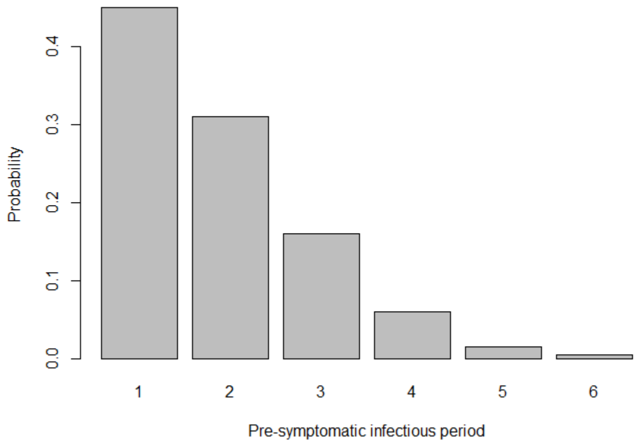

Fig. S11 Distribution for pre-symptomatic period in days [6].

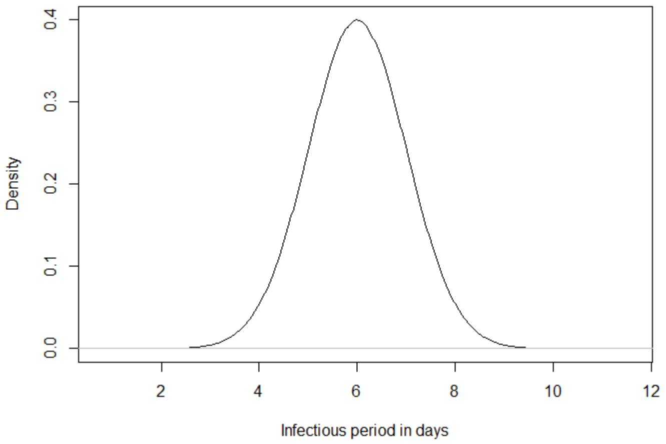

Fig. S12 Distribution for infectious period [6].

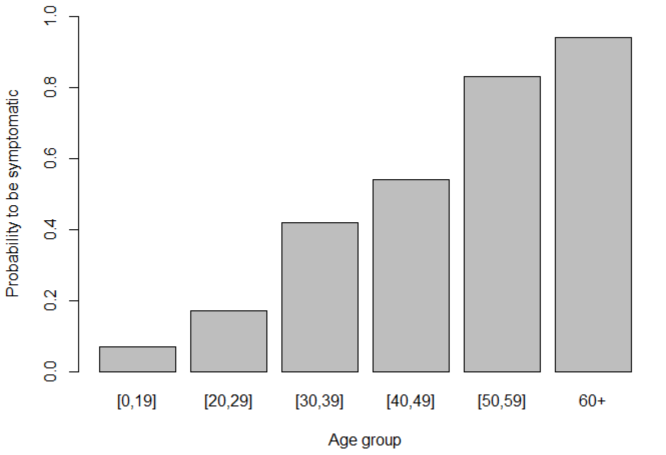

Fig. S13 Probability to be symptomatic by age group (Willem at al., 2020) [6]

9 Disease-related mortality

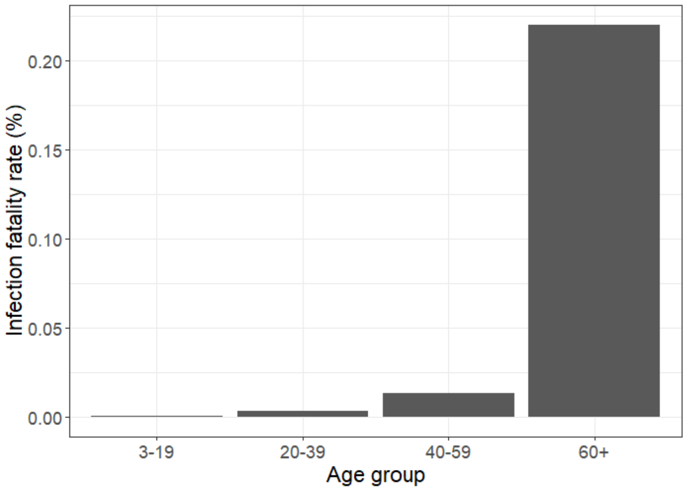

Fig. S14 Infection fatality rates for 2009 (H1N1) pandemic influenza in Hong Kong by age group [14].

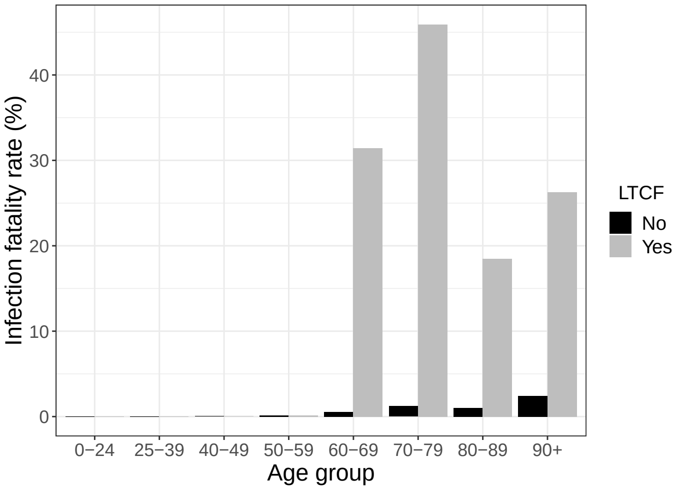

Fig. S15 Infection fatality rates for COVID-19 in Belgium by age group and household type (LTCF or non-LTCF) [15].

10 Estimation of QALY losses

We estimate the QALYs lost due to premature death at age x, $1 \leq x < \omega$, as follows [16]:

$$dQALY(x) = \frac{\sum_{u=x}^{\omega} L_s(u) \cdot Q(u) \cdot qCM \cdot (1+r)^{-(u-x)}}{l_s(x)}, \tag{3}$$

where

$$l_s(x) = 100,000 \cdot \prod_{a=1}^{x} e^{-d(a) \cdot SMR} \tag{4}$$

with $L_s(x)$ being the average of $l_s(x)$ and $l_s(x+1)$ and $Q(x)$ denotes the population average quality of life tariff at age x for Belgium in 2018 [17]. The parameter qCM adjust the quality of life for the impact of pre-existing comorbidity, while the impact of comorbidity on the risk of dying is summarised in the standardised mortality ratio (SMR). Finally, the instantaneous death rate is denoted $d(x)$ and r is the discount rate applied to incorporate the assumption that current health benefits are valued higher than future health benefits. As in [16], we assume that $SMR = 1.5$ and $qCM = 90\%$.

11 Additional results

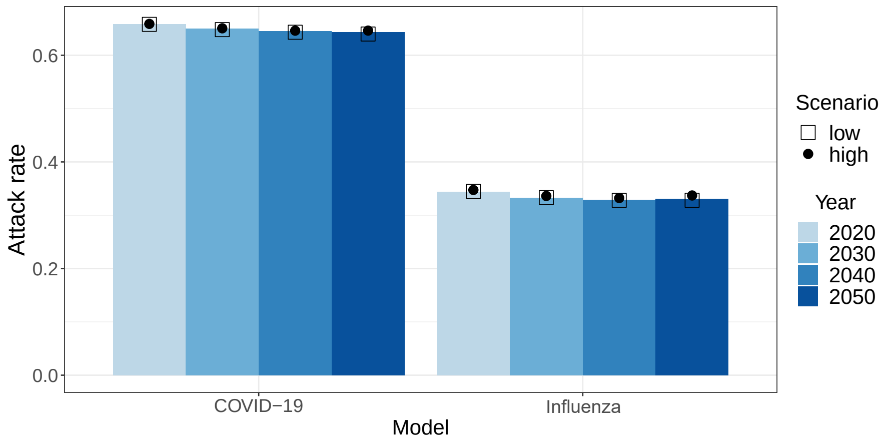

Fig. S16 Mean attack rate in total population by simulation year, model and demographic scenario.

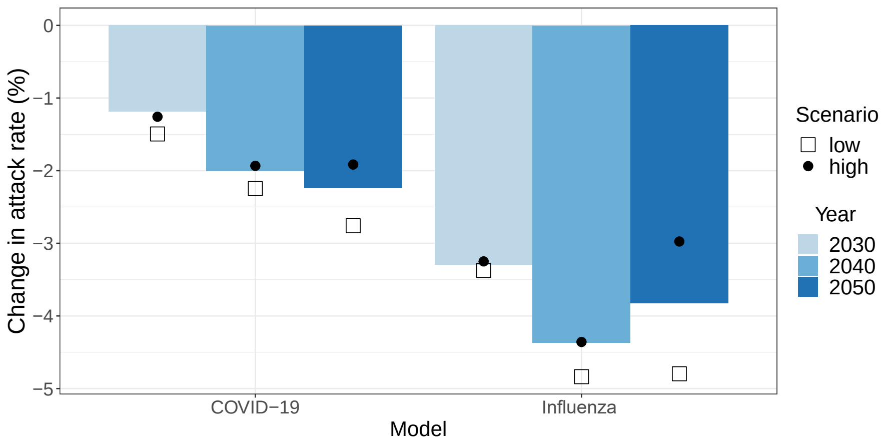

Fig. S17 Change in overall attack rates relative to 2020.

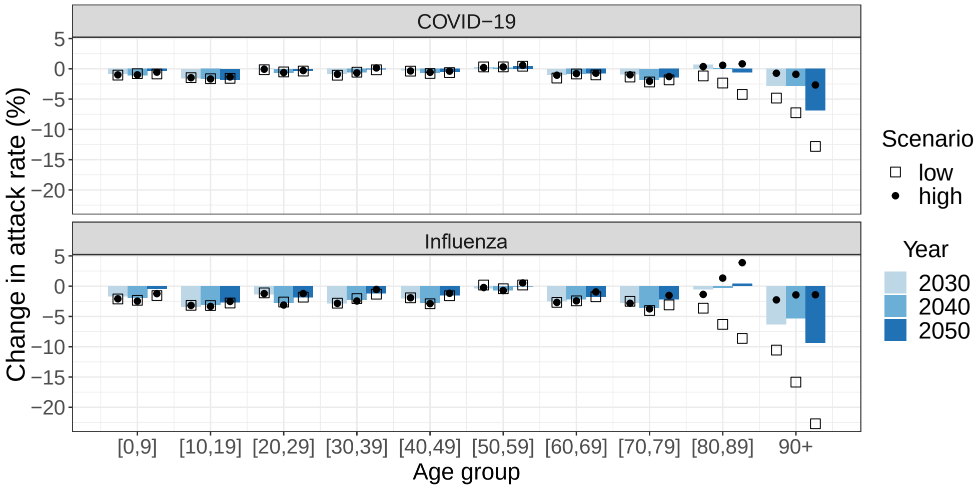

Fig. S18 Change in age-specific attack rates relative to 2020.

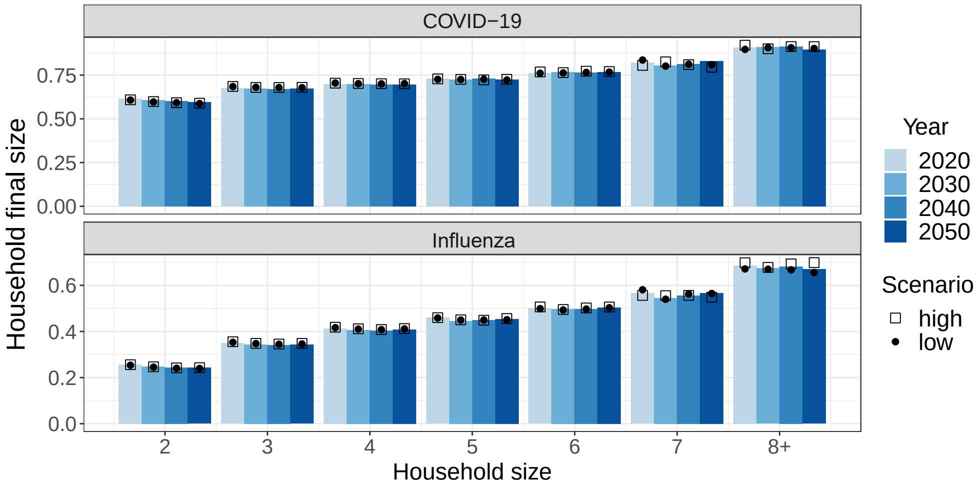

Fig. S19 Mean number of infected household members as proportion of household size by year, model and demographic scenario.

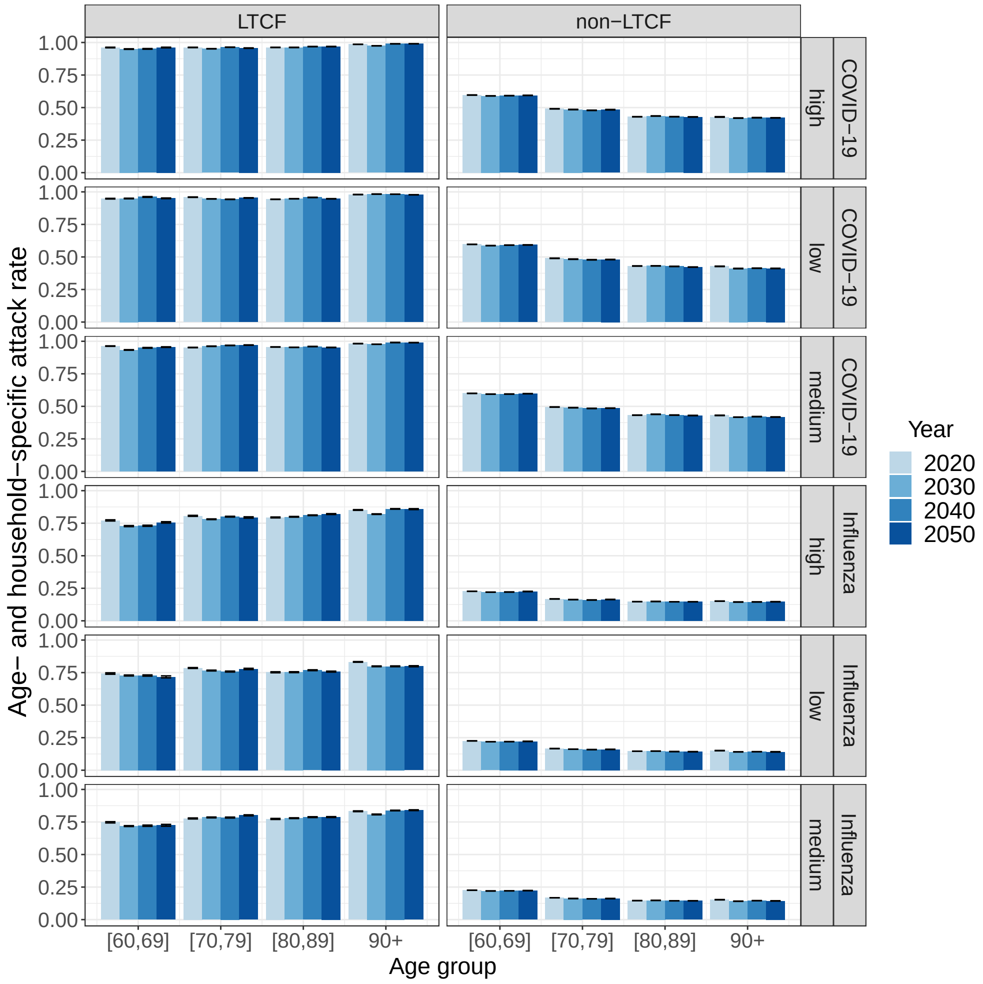

Fig. S20 Age and household-specific attack rate in the elderly population. Columns: Household type. Rows: Model and demographic scenario.

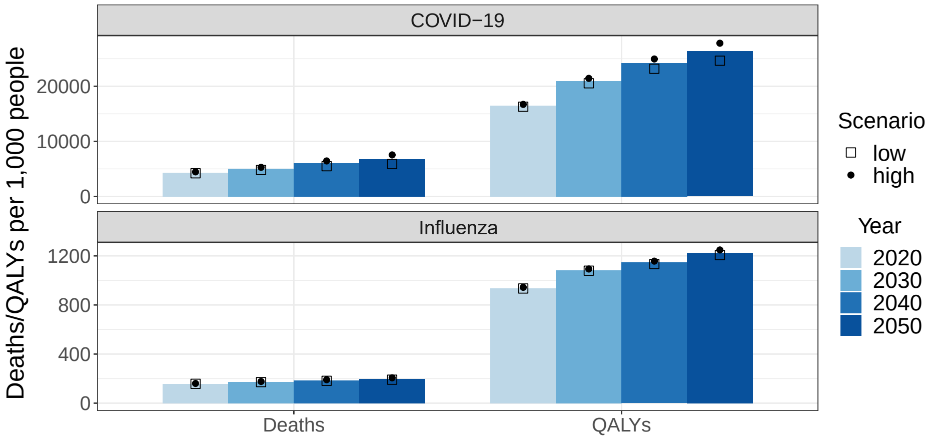

Fig. S21 Disease-related deaths and QALY losses per 1,000 people in the population. Upper panel: COVID-19, lower panel: ILI.

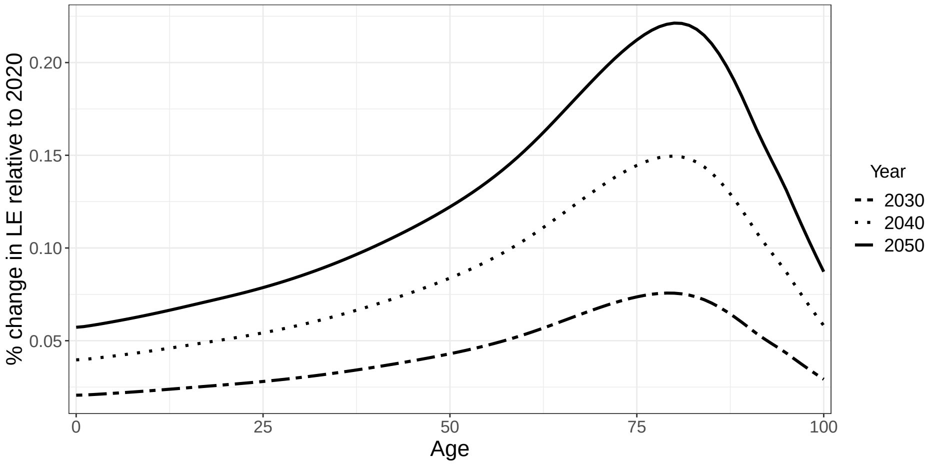

Fig. S22 Relative change in life expectancy across ages compared to 2020 (Statbel).

References

[1] Hoang, T.V., Coletti, P., Kifle, Y.W., Kerckhove, K.V., Vercruysse, S., Willem, L., Beutels, P., Hens, N.: Close contact infection dynamics over time: insights from a second large-scale social contact survey in Flanders, Belgium, in 2010-2011. BMC Infectious Diseases **21**(1), 1–15 (2021) https://doi.org/10.1186/s12879-021-05949-4

[2] Willem, L., Van Hoang, T., Funk, S., Coletti, P., Beutels, P., Beutels, P., Hens, N., Hens, N.: SOCRATES: An online tool leveraging a social contact data sharing initiative to assess mitigation strategies for COVID-19. BMC Research Notes **13**(1), 1–8 (2020) https://doi.org/10.1186/s13104-020-05136-9

[3] Ball, F., Mollison, D., Scalia-Tomba, G.: Epidemics with Two Levels of Mixing. The Annals of Applied Probability **7**(1), 46–89 (1997)

[4] Abrams, S., Wambua, J., Santermans, E., Willem, L., Kuylen, E., Coletti, P., Libin, P., Faes, C., Petrof, O., Herzog, S.A., Beutels, P., Hens, N.: Modelling the early phase of the Belgian COVID-19 epidemic using a stochastic compartmental model and studying its implied future trajectories. Epidemics **35**, 100449 (2021) https://doi.org/10.1016/j.epidem.2021.100449

[5] Coletti, P., Libin, P., Petrof, O., Willem, L., Abrams, S., Herzog, S.A., Faes, C., Kuylen, E., Wambua, J., Beutels, P., Hens, N.: A data-driven metapopulation model for the Belgian COVID-19 epidemic: assessing the impact of lockdown and exit strategies. BMC Infectious Diseases **21**(1), 1–12 (2021) https://doi.org/10.1186/s12879-021-06092-w

[6] Willem, L., Abrams, S., Libin, P.J.K., Coletti, P., Kuylen, E., Petrof, O., Møgelmose, S., Wambua, J., Herzog, S.A., Faes, C., Beutels, P., Hens, N.: The impact of contact tracing and household bubbles on deconfinement strategies for COVID-19. Nature Communications **12**(1), 1–9 (2021) https://doi.org/10.1038/s41467-021-21747-7

[7] Biggerstaff, M., Cauchemez, S., Reed, C., Gambhir, M., Finelli, L.: Estimates of the reproduction number for seasonal, pandemic, and zoonotic influenza: A systematic review of the literature. BMC Infectious Diseases **14**(1), 1–20 (2014) https://doi.org/10.1186/1471-2334-14-480

[8] Cowling, B.J., Lau, M.S.Y., Ho, L.M., Chuang, S.K., Tsang, T., Liu, S.H., Leung, P.Y., Lo, S.V., Lau, E.H.Y.: The effective reproduction number of pandemic influenza: Prospective estimation. Epidemiology **21**(6), 842–846 (2010) https://doi.org/10.1097/EDE.0b013e3181f20977

[9] Petersen, E., Koopmans, M., Go, U., Hamer, D.H., Petrosillo, N., Castelli, F., Storgaard, M., Al Khalili, S., Simonsen, L.: Comparing SARS-CoV-2 with SARS-CoV and influenza pandemics. The Lancet Infectious Diseases **20**(9), 238–244

(2020) https://doi.org/10.1016/S1473-3099(20)30484-9

[10] Casado, I., Martínez-Baz, I., Burgui, R., Irisarri, F., Arriazu, M., Elía, F., Navascués, A., Ezpeleta, C., Aldaz, P., Castilla, J., Abad, I., Agreda, J., Álvarez, E., Arana, J.J., Arceiz, I., Arina, E., Artajo, M.D., Arza, A., Ayerdi, K., ..., Castilla, J.: Household transmission of influenza A(H1N1)pdm09 in the pandemic and post-pandemic seasons. PLoS ONE **9**(9) (2014) https://doi.org/10.1371/journal.pone.0108485

[11] Madewell, Z.J., Yang, Y., Longini, I.M., Halloran, M.E., Dean, N.E.: Household Secondary Attack Rates of SARS-CoV-2 by Variant and Vaccination Status: An Updated Systematic Review and Meta-analysis. JAMA Network Open **5**(4), 229317 (2022) https://doi.org/10.1001/jamanetworkopen.2022.9317

[12] Mondiale, O.: New influenza A (H1N1) virus : global epidemiological situation, June 2009 = Nouveau virus grippal A (H1N1) : situation épidémiologique mondiale, juin 2009. Weekly Epidemiological Record = Relevé épidémiologique hebdomadaire **84**(25), 249–257 (2009)

[13] He, X., Lau, E.H.Y., Wu, P., Deng, X., Wang, J., Hao, X., Lau, Y.C., Wong, J.Y., Guan, Y., Tan, X., Mo, X., Chen, Y., Liao, B., Chen, W., Hu, F., Zhang, Q., Zhong, M., Wu, Y., Zhao, L., Zhang, F., Cowling, B.J., Li, F., Leung, G.M.: Temporal dynamics in viral shedding and transmissibility of COVID-19. Nature Medicine **26**(5), 672–675 (2020) https://doi.org/10.1038/s41591-020-0869-5

[14] Riley, S., Kwok, K.O., Wu, K.M., Ning, D.Y., Cowling, B.J., Wu, J.T., Ho, L.M., Tsang, T., Lo, S.V., Chu, D.K.W., Ma, E.S.K., Peiris, J.S.M.: Epidemiological characteristics of 2009 (H1N1) pandemic influenza based on paired sera from a longitudinal community cohort study. PLoS Medicine **8**(6) (2011) https://doi.org/10.1371/journal.pmed.1000442

[15] Molenberghs, G., Faes, C., Verbeeck, J., Deboosere, P., Abrams, S., Willem, L., Aerts, J., Theeten, H., Devleesschauwer, B., Sierra, N.B., Renard, F., Herzog, S., Lusyne, P., Van Der Heyden, J., Van Oyen, H., Van Damme, P., Hens, N.: COVID-19 mortality, excess mortality, deaths per million and infection fatality ratio, Belgium, 9 March 2020 to 28 June 2020. Eurosurveillance **27**(7), 1–10 (2022) https://doi.org/10.2807/1560-7917.ES.2022.27.7.2002060

[16] Briggs, A.H., Meacock, R., Goldstein, D.A., Kirwin, E., Wisløff, T.: Estimating (quality-adjusted) life-year losses associated with deaths: With application to COVID-19. Health Economics **30**, 699–707 (2022) https://doi.org/10.1002/hec.4208

[17] Van Wilder, L., Charafeddine, R., Beutels, P., Bruyndonckx, R., Cleemput, I., Demarest, S., De Smedt, D., Hens, N., Scohy, A., Speybroeck, N., Heyden, J., Yokota, R.T.C., Van Oyen, H., Bilcke, J., Devleesschauwer, B.: Belgian population norms for the EQ-5D-5L, 2018. Quality of Life Research **31**(2), 527–537 (2022)

https://doi.org/10.1007/s11136-021-02971-6
